# New Strategies Protecting from Ischemia/Reperfusion

**DOI:** 10.3390/ijms232415867

**Published:** 2022-12-14

**Authors:** Thierry Hauet, Didier F. Pisani

**Affiliations:** 1 INSERM U1313, IRMETIST, Université de Poitiers et CHU de Poitiers, 86021 Poitiers, France; 2 Université Côte d’Azur, CNRS, LP2M, 06108 Nice, France

This Special Issue aims to highlight new avenues in the management of Ischemia/Reperfusion (I/R) injury. I/R are the most common causes of debilitating diseases and death in stroke, cardiovascular ischemia, organ transplantation, etc. I/R is due to a partial or complete arrest of blood flow, characterized by a noticeable drop in available oxygen coupled to the deprivation of energy substrates, and then by an abrupt increase in the oxygen supply of the affected region, both inducing critical damage to cells and tissues. In recent decades, significant advances in the comprehension of the biological and molecular pathways managing the effects of I/R have been undertaken. Nevertheless, no clinical protocol or pharmacological approach is completely satisfactory and effective in protecting tissues and organs from the deleterious consequences of I/R. I/R management is a complex area that depends on the situation. In the case of surgical intervention with circulation arrest, as for heart surgery or organ transplantation, I/R are predictable and can thus be managed prior to the arrest of blood flow with preconditioning protocols or during the intervention with per-conditioning protocols [1]. Delayed cerebral ischemia after hemorrhage is similar to these ischemia and takes places several days after the aneurysm [2]. These predictabilities allow the development of numerous protocols with different treatment windows [1]. In this issue, some examples of original protocols are displayed from long-term/multiple-dose (ten days) [3] to acute/single-dose preconditioning [4,5], as well as the management of the ischemic phase using temperature control [6].

Differently, myocardial infarction, stroke or testicular torsion are sudden, and the management can only occur during ischemia [7] and during blood reflow [8]. Despite the identification of many protective treatments in experimental models of I/R, their translation to the clinic has shown very disappointing benefits for patients, especially for stroke and infarct [9,10]. This seems to be due, on the one hand, to the multifactorial character of I/R and on other hand to the lack of standardized procedures for preclinical study, allowing a better comprehension and comparison of the numerous results published every year. A decade ago, a standardized protocol for neuroprotective clinical study was proposed, leaning on centers of reference and care networks [11]. Inspired by these clinical “STAIR” recommendations, Haupt and collaborators discussed in this Special Issue the different animal models mimicking human stroke, the time of treatment and the kind of analysis, with the objectives of proposing a new standardized issue for stroke preclinical model study [8].

Independent of its origin and the affected organ, I/R leads to various deleterious processes such as oxidative stress, energy distress, endoplasmic reticulum stress, acidification and inflammation, along with the production of stress mediators, including cytokines, fibrotic factors, oxygen and nitrogen species, these last include peroxynitrite, which is one of the more deleterious mediators during I/R [1,12]. The multiplicity of these processes involves numerous cellular mechanisms and pathways, which are all potential therapeutic targets. In this Special Issue, original approaches targeting various ubiquitous kinase pathways are proposed for testicular torsion and kidney transplantation [3,4,7], which could be extended to other ischemic events, as well as specific combinatory treatment using hormones in the case of uterus transplantation [5]. In the majority of ischemic situations, mitochondria play a central role as depletion in oxygen supply leads to the impairment of mitochondrial function and loss of energy metabolites, mainly following at the time of reperfusion by oxidative stress and disturbance of Ca^2+^ homeostasis crucial for cell viability [1,13]. Thus, the majority of anti-ischemic strategies targets, directly or indirectly, mitochondria function, integrity or by-products, as displayed in the different articles of this Special Issue.

In conclusion, I/R management is a crucial field for human health, and we must continue our efforts to decipher the mechanisms of cell defense and/or cell sensitivity to this oxygen supply disturbance. Furthermore, it will be crucial for all scientists working in this field to use more standardized procedures, to consider the specificity and complexity of the organs and finally to integrate the particularity of the human situation for better success in clinical application. We hope this Special Issue may have contributed to improving our knowledge about the diversity of I/R mechanisms, problems and management.
